# Oxygen and Nitrogen Transfer in Furnaces in Crystal Growth of Silicon by Czochralski and Directional Solidification Processes

**DOI:** 10.3390/ma15051843

**Published:** 2022-03-01

**Authors:** Koichi Kakimoto, Xin Liu, Satoshi Nakano

**Affiliations:** Research Institute for Applied Mechanics (RIAM), Kyushu University, Fukuoka 816-8580, Japan; liuxin@riam.kyushu-u.ac.jp (X.L.); snaka@riam.kyushu-u.ac.jp (S.N.)

**Keywords:** simulation, silicon, impurity, transfer, crystal growth

## Abstract

Impurity concentrations of oxygen, carbon, nitrogen, iron, and other heavy metals should be well controlled in silicon crystals to maintain the crystal quality for application in electronic and solar cell devices. Contamination by impurities occurs during the melting of raw materials and during the crystal growth process. Quantitative analysis of impurity transfer using numerical and experimental analysis is important to control impurity concentrations. This paper reviews the analysis of the impurity transport phenomena in crystal growth furnaces of Czochralski and directional solidification methods by a model of global analysis and an experiment during the crystal growth of silicon.

## 1. Introduction

Internet society requires electronic devices such as memory and logic of high quality. They need silicon (Si) wafers with a reasonable concentration of oxygen (O) [1]. The power devices also need Si wafers with low carbon (C) and O concentrations [2]. Solar cells made by Si crystals also need wafers with low concentrations of O and C to boost conversion efficiency [3]. The carrier lifetime in Si crystals plays an important role in the device process of power devices as well as solar cells. O precipitates and the C concentration degrade the lifetime of carrier in bulk Si crystals [4]. Therefore, the production of Si wafers with a long lifetime required the reduction of concentrations of O and C impurities during the crystal growth process of the Czochralski growth of silicon (CZ-Si). Carbon monoxide (CO) generation on the graphite components and the interface between quartz (SiO_2_) and graphite [5,6,7,8] in a furnace cause contamination of C in Si crystals. It is a key point to control CO generation and C incorporation from the melting period of the feedstock to the final stage of the crystal growth process. 

The transport of C and O, as well as the heavy metals during the CZ-Si crystal growth, have been investigated over the last few decades [5,9,10,11,12,13,14]. Vorob’ev et al. [13] reported the calculated distribution of oxygen concentration in a crystal grown by the directional solidification method which is similar to that of the other published paper [9]. Trempa et al. [14] reported the experimental data of carbon and nitrogen as well as SiC and Si_3_N_4_ precipitates in a grown crystal obtained from the directional solidification method. They discussed the details of a formation mechanism on SiC and Si_3_N_4_ precipitates during crystal growth. Back diffusion of CO generated by the carbon heater has been found to occur. The interface between the crucible made of SiO_2_ and the carbon holder is one of the causes of C contamination in the Si melt and raw material. The condensation mechanism of C in the melt of Si should be studied based on a time-dependent study, such as a numerical method, or by in situ observation during the CZ-Si crystal growth. A global model containing a chemical reaction including CO and silicon monoxide (SiO) based on a thermodynamic analysis of the reactions at the elevated temperature was reported by Bornside et al. [5] A coupled model with a transport model for predicting SiO and CO concentrations in gas of argon (Ar), and C and O concentrations in the Si melt was reported by Gao et al. [6]. They reported the results by suing a quasi-static method of calculation. Such a method cannot take into account C accumulation during CZ-Si crystal growth [6], then the quasi-static assumption is not able to predict the C content in Si melt. Then, the transient method of heat and mass transport is necessary to study C condensation quantitatively during the melting process of CZ-Si crystal growth [15].

For the directional solidification method (DS) of multicrystalline Si, the top part of the ingot has a large number of grains. The conversion efficiency is degraded by the grains. The grain boundaries formed in Si crystals have high concentrations of SiC and Si_3_N_4_ as Arafune et al. reported [3]. The SiC and Si_3_N_4_ causes the grains. Lu et al. reported that Si_3_N_4_ reacted with O in the Si melt, then formed Si oxynitrides in polycrystalline sheet Si [16]. The results informed us that Si oxynitrides are concentrated in the Si crystal. Then, it is important to investigate the mechanism of Si oxynitride formation using numerical analysis by taking into account a phase diagram of the Si(l)–N–O system [17].

This review paper reports the contamination mechanism of O and C during Si crystal growth in a furnace. Moreover, numerical results using a ternary Si(l)–N–O system were introduced to investigate the formation of Si_3_N_4_ and Si_2_N_2_O during the crystal growth process.

## 2. Experimental and Numerical Methods

### 2.1. Czochralski Method

**Figure 1:**
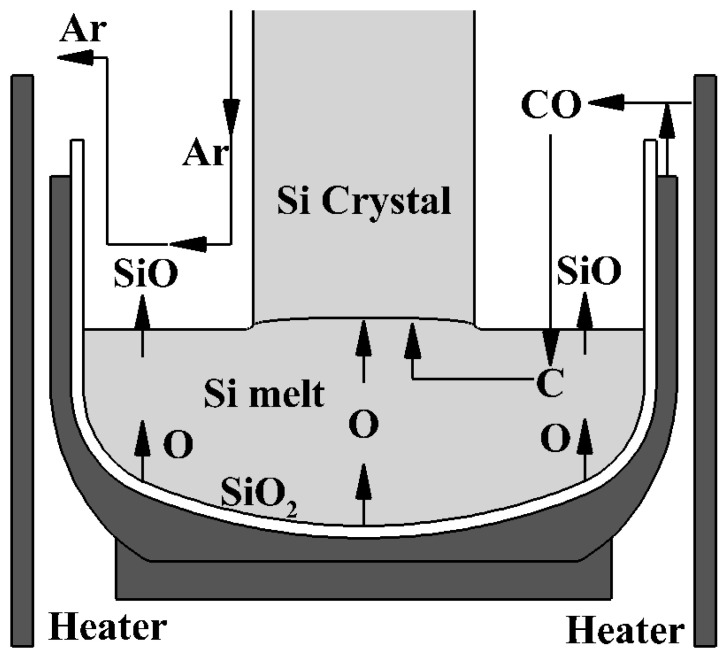
Schematic diagram of SiO and CO transfer in a CZ furnace.

**Figure 2:**
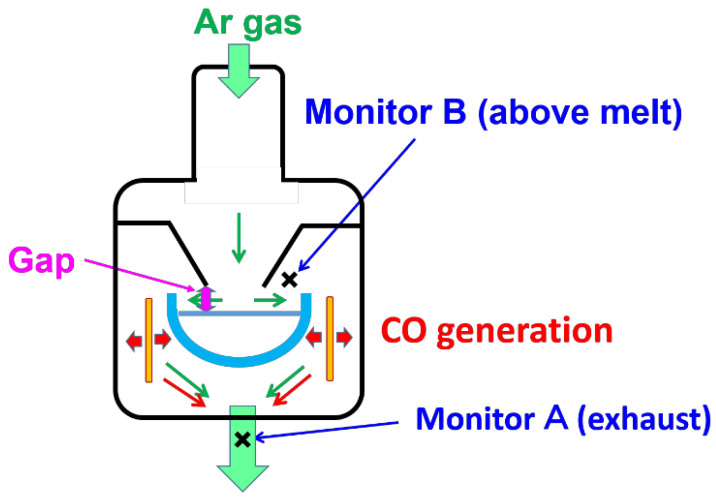
The monitoring locations detecting the CO concentration. The locations, A and B, were near the melt and the exhaust, respectively. Reproduced with permission from Y. Miyamura, H. Harada, X. Liu, S. Nakano, S. Nishizawa, K. Kakimoto, Journal of Crystal Growth; published by Elsevier, 2019 [8].

A schematic of C and O transfer in a CZ furnace is shown in Figure 1 [8]. Miyamura et al. reported an experimental procedure of the direct measurement of CO concentration in a CZ furnace applying a gas detection method (GC-400 PLUS C IF) [8]. The measurement system is shown in Figure 2 at monitors A and B. Temperature at an analyzer was room temperature, and the measurement was made via quasi in situ observation. The authors set sampling points at the locations near the Si melt and at the exhaust of Ar in CZ furnace, indicated by A and B, respectively. A total of 25 kg of polycrystalline Si was melted in a quartz (SiO_2_) crucible. The surface of the heater and the interface between the SiO_2_ crucible and carbon susceptor generated CO gas. 

Several papers [18,19] reported that O dissolved from the SiO_2_ crucible, then SiO was formed in the melt during the melting process. The papers also reported that CO was generated by the SiO at the surface of the heater and components made of carbon, and the CO was accumulated in the Si melt. This experiment had no growing crystals due to the main contamination is the melting process. The authors measured the CO concentration under a constant temperature. To study the effect on CO contamination of the melt, they changed the total pressure and gas flow rate of Ar in the growth furnace from 1.599 × 10^3^ to 18.665 × 10^3^ Pa and 20 to 80 SLPM (standard liter per minute at 273.15 K and 1.0135 × 10^4^ Pa), respectively. The gap width between the melt and heat shield was also changed from 20 to 60 mm to determine the effect on CO contamination.

**Figure 3:**
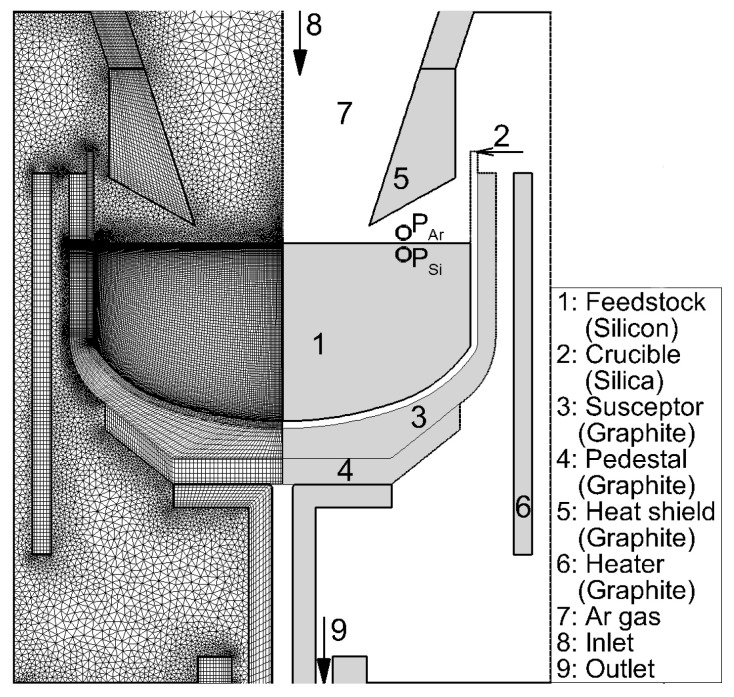
Geometry of the CZ-Si crystal growth. Reproduced with permission from X. Liu, B. Gao, S. Nakano, K. Kakimoto, Journal of Crystal Growth; published by Elsevier, 2017 [20].

Liu et al. reported numerical results with the same furnace configuration [20,21]. They took into account the calculations of temperature distribution in the components, interface shape between the melt and a crystal, convection, and impurity transport, and convection of Ar gas and chemical products in the gas. They used the furnace, and the grids for the CZ-Si crystal growth is shown in Figure 3. 

Figure 3 includes feedstock (1), the quartz crucible (2), the suscepter (3), pedestal (4), and heater (6) made of graphite. The figure also includes heat shields (5). Ar gas (7) was introduced from the top (8) and purged from the bottom (9) of the furnace, as shown in Figure 3. The algorithms for the heat transfer have been reported in some papers [22,23]. 

### 2.2. Directional Solidification Method

**Figure 4:**
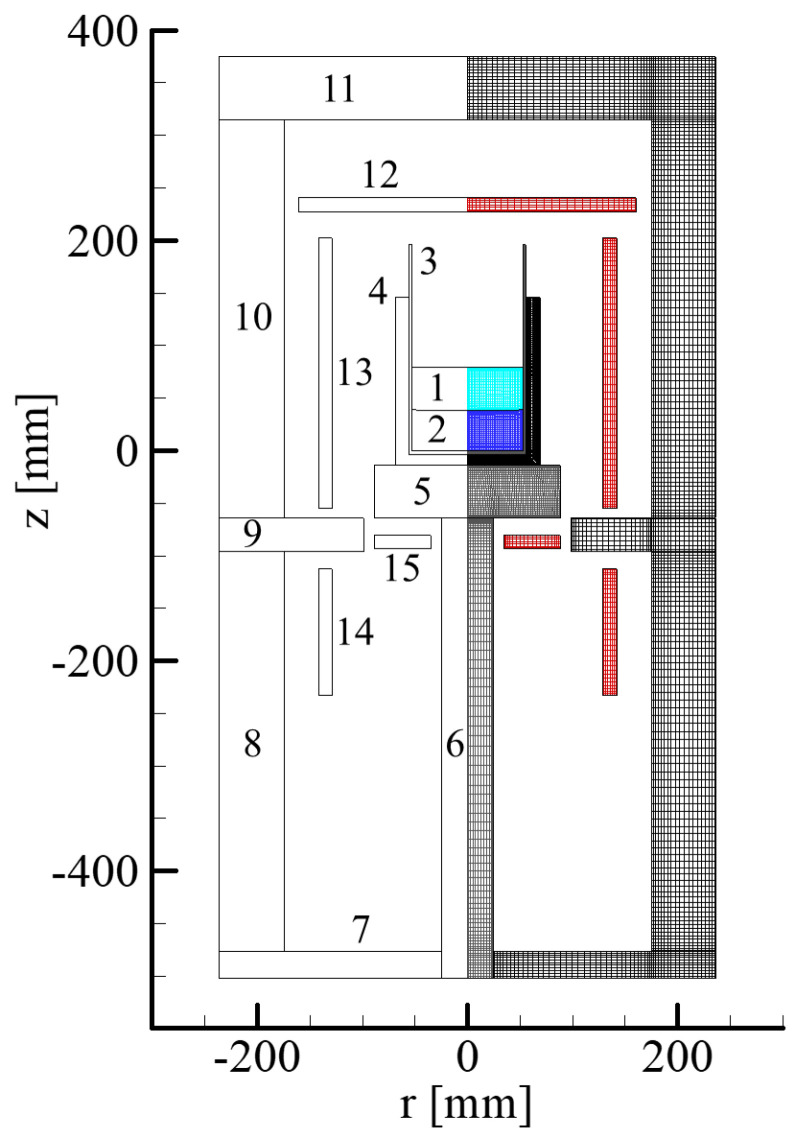
Configuration and domain partition of the furnace. 1: melt, 2: crystal, 3–4: crucibles, 5–6: pedestals, 7–11: heat shields, 12–15: heaters Reproduced with permission from S. Hisamatsu, H. Matsuo, S. Nakano, K. Kakimoto, Journal of Crystal Growth; published by Elsevier, 2009, [17].

Hisamatsu et al. [16] reported that the O and nitrogen (N) concentrations, as well as the formation of SiO_2_, Si_3_N_4_, and Si_2_N_2_O in crystal growth process. They used a model of DS furnace with computational grid shown in Figure 4. 

## 3. Results

### 3.1. Czochralski Method

**Figure 5:**
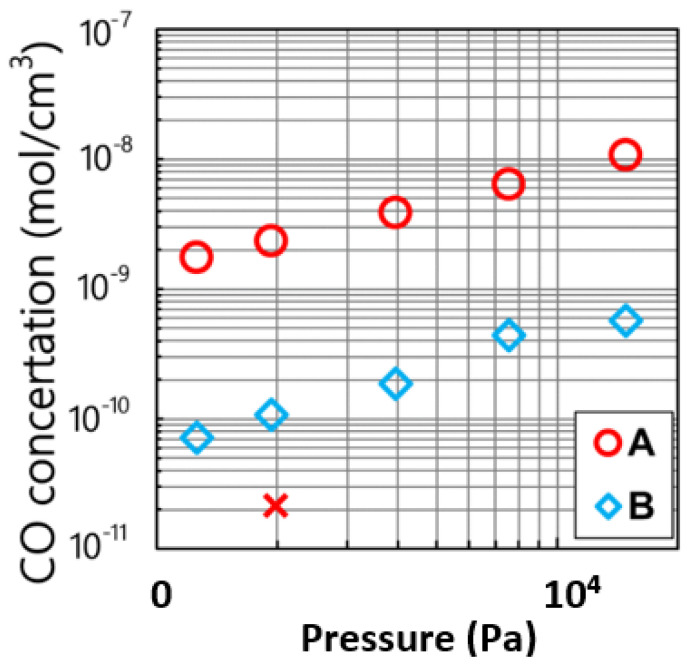
CO concentration as a function of Ar gas pressure. Monitor points A and B correspond to those in Figure 2. The cross indicates the measured concentration obtained without the Si melt and with a quartz crucible at A Reproduced with permission from Y. Miyamura, H. Harada, X. Liu, S. Nakano, S. Nishizawa, K. Kakimoto, Journal of Crystal Growth; published by Elsevier, 2019 [8].

Miyamura et al., reported the pressure dependence of the CO concentrations at A and B, shown in Figure 5. The cross shows the concentration measured at A. They reported that the concentration of CO caused by the reaction between the Si melt and the SiO_2_ crucible was higher value than that caused by the SiO_2_ crucible without Si melt. This result shows that SiO, which was generated through the dissolution of the SiO_2_ crucible by the Si melt, was the one of the important reactants in the generation of CO. The values measured at monitors A and B increased linearly. It was concluded that the measured CO concentrations increased proportionally with the total pressure in the furnace. 

In general, flow velocity of Ar gas decreased as a function of pressure due to the ideal gas law. CO caused by the graphite diffused back as the gas velocity in the furnace decreased. Thus, the decrease in the velocity enhances the back diffusion of CO toward the gas and the melt interface. Then, it was concluded that the CO concentration increased as a function of the total pressure in the furnace. The paper reported that the CO concentration at B was smaller than that at A. CO concentration at A increased due to CO transport from the heater to the exhaust.

**Figure 6:**
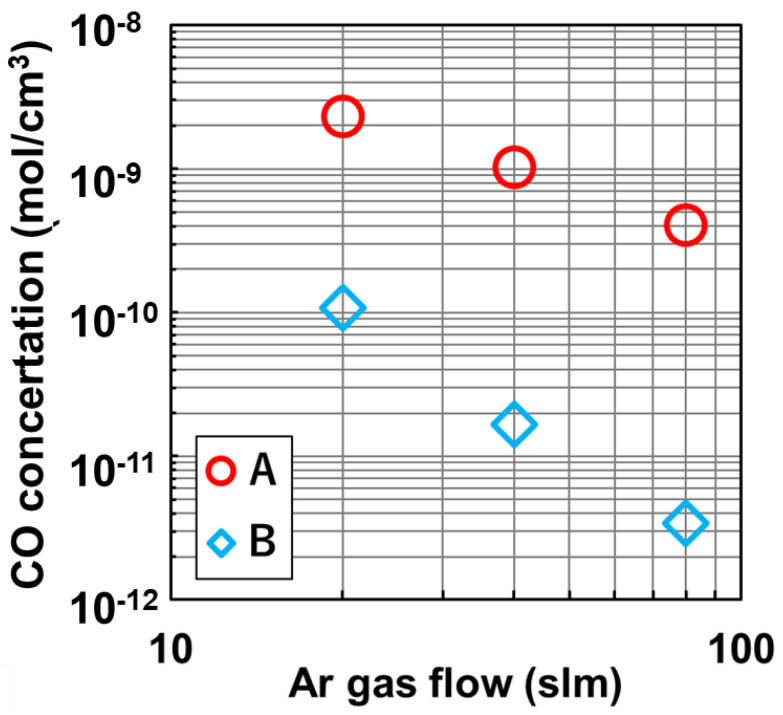
Measured CO concentration as a function of Ar gas flow rate in the furnace Reproduced with permission from Y. Miyamura, H. Harada, X. Liu, S. Nakano, S. Nishizawa, K. Kakimoto, Journal of Crystal Growth; published by Elsevier, 2019 [8].

The paper by Miyamura et al. reported [8] that the measured CO concentrations at A and B as a function of the flow rate of Ar gas in the furnace as shown in Figure 6. The CO concentration decreased as the flow rate of Ar gas increased. At monitor A, the CO concentration changed, which was greater than the value shown in Figure 5. The results show that the gas flow rate can modify CO concentration effectively by modification of the pressure of Ar in the furnace. The correlation between the pressure and gas flow rate on CO concentration were also reported in the previous numerical studies [13]. The paper by Miyamura et al. [8] concluded that the decrease in the CO concentration at A and B as a function of the gas flow rate was based on the higher Péclet number. The higher flow rate of gas also removed SiO from the furnace effectively, which was one of the reactants for the production of CO [8]. The small gap enhances the flow velocity of Ar gas.

**Figure 7:**
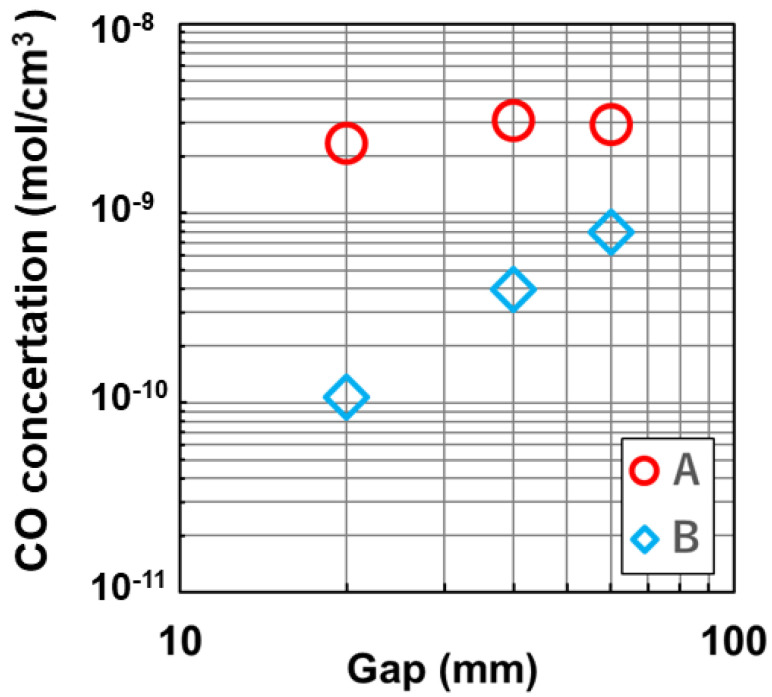
Measured CO concentration as a function of gap width between the melt surface and thermal shield. Reproduced with permission from Y. Miyamura, H. Harada, X. Liu, S. Nakano, S. Nishizawa, K. Kakimoto, Journal of Crystal Growth; published by Elsevier, 2019 [8].

The paper [8] reported the measured CO concentrations at A and B as a function of the gap width between the melt surface and thermal shield shown in Figure 7 [8]. The concentration at A was almost constant as a function of gap width, whereas the concentration at B increased with a wide gap width. The CO concentration at monitor A remained constant because of the constant production of CO at the surface of the heater. The increase of CO concentration at B was originated by a large Péclet number, which is a ration between the flow velocity of Ar gas and the diffusion constant of CO in Ar gas. The large flow velocity at monitor A swept out CO gas returned from inside of the furnace. Then, the concentration of CO at monitor A was almost constant. When the gap width was small, the flow velocity of the Ar gas increases which induced flow instability or changing direction of flow of the melt. Then, oxygen concentration in the melt changes. The operator of crystal growth should control the flow velocity not to be unstable by optimizing the gap width. Concerning the effect of changing distance between the crucible (no. 2) and thermal shield (no. 5) on Co transfer, the distance is also important to control CO concentration. Meanwhile, the quartz crucible becomes soft during crystal growth, then the distance chances as a function of duration time of crystal growth. Then, it is difficult to discuss the effect qualitatively. 

The heat shield (no. 5) shown in Figure 3 is another source of CO generation. Some crystal growers coated the shield by SiC to prevent the generation of CO gas. 

### 3.2. Directional Solidification Method

**Figure 8:**
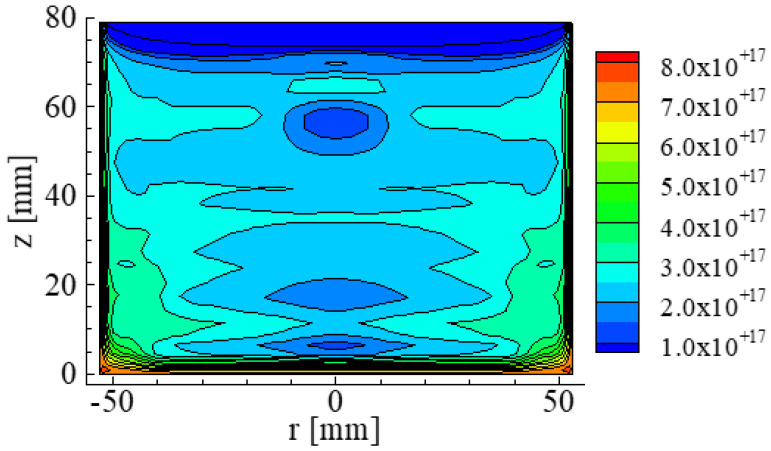
Concentration distribution of O in a Si crystal and the melt. The unit of concentration is atoms/cm^3^. Reproduced using data from reference [17].

The paper reported by Hisamatsu et al., [17] reports that the distribution of O concentration in the Si crystal and the melt before the final stage of crystallization shown in Figure 8. The O concentration was large near the crucible and decreased toward the top area of the crystal. Other papers [13,14] reported that the O concentrations were approximately the same level as those reported by the paper reported by Hisamatsu et al. [17] 

**Figure 9:**
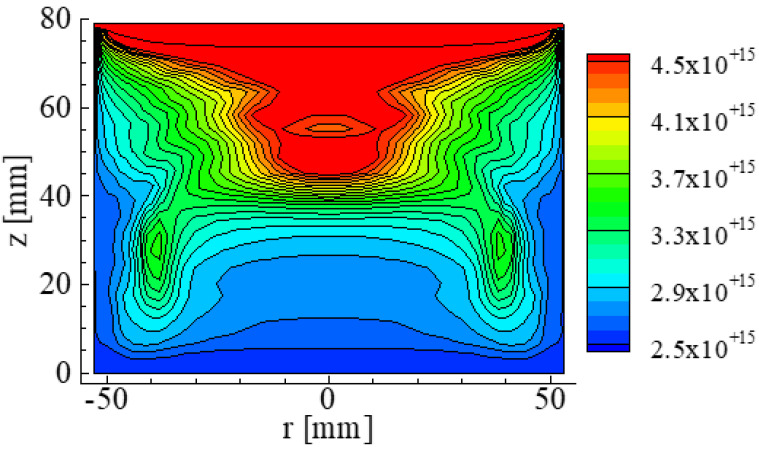
Concentration distribution of N in an Si crystal and melt. The unit of concentration is atoms/cm^3^ Reproduced using data from reference [17].

The paper also reported the concentration distribution of N in the crystal and the melt shown in Figure 9 [17]. The N concentration near the top of the crystal was 4.5 × 10^15^ atoms/cm^3^. The value is almost the same as the solubility limit of N in the Si crystal. The results of the calculations were similar to the values of 1 × 10^15^ to 7 × 10^15^ atoms/cm^3^ [11].

**Figure 10:**
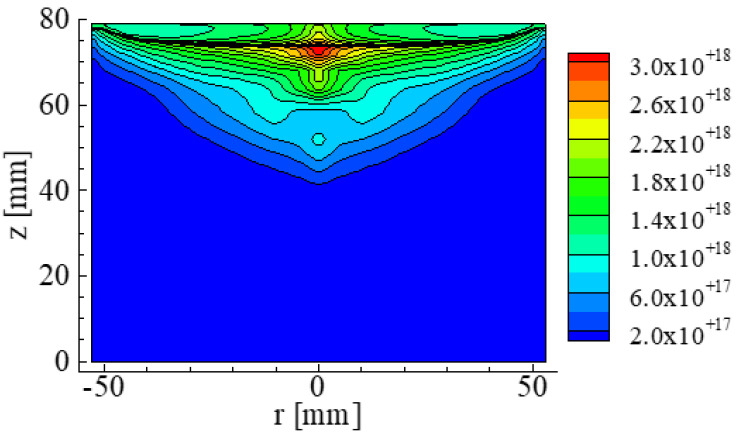
Concentration distribution of Si_3_N_4_ in Si. The unit of concentration is atoms/cm^3^ Reproduced using data from reference [17].

The paper included the concentration distribution of Si_3_N_4_ in the Si crystal and the melt shown in Figure 10. The reacted Si_3_N_4_ condensed near the top of the Si crystal. Additionally, it was also reported that Si_3_N_4_ condensed at an initial stage of the crystallization process in higher concentrations. The calculation results are similar to the result of study on Si_3_N_4_ [23]. 

**Figure 11:**
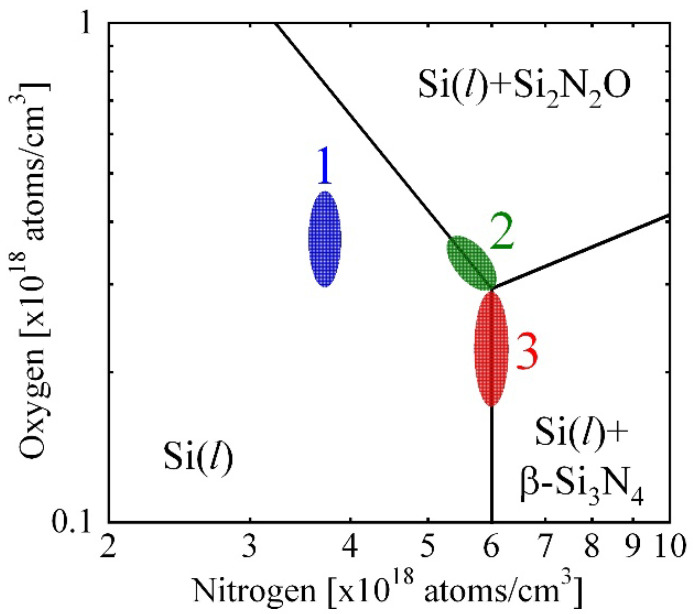
Schematic diagram of the reaction of Si_3_N_4_ and Si_2_N_2_O as a function of O and N concentrations in the Si melt in the Si(l)–N–O phase diagram. Numbers 1,2 and 3 denotes in the area Si(l), the boundary between Si(l) and Si(l) + Si_2_N_2_O and the boundary between Si(l) + Si_2_N_2_O and Si(l) + Si_3_N_4_. Reproduced using data from reference [17].

Hisamatsu et al. [17] estimated that the area of this phenomena was located in region 1 of Figure 11. O evaporated from the top of the melt [18], the O concentration at the top of the melt decreased with an increase in crystal growth process. Conversely, N concentration in the melt increased as a function of crystal growth duration time due to the equilibrium segregation coefficient [12]. As a result, the O and N concentrations at the melt/crystal interface shifted from 1 to 2 in Figure 11. Then, Si_2_N_2_O was condensed at the initial time of the process. When the crystal was solidified further, Si_3_N_4_ condensed shown in area 3 in Figure 11. The distribution of N in the crystal, especially at the top of the crystal is governed by the phase diagram shown in Figure 11 meaning that N is changed to Si_2_N_2_O as the concentration was increased at the top pf the crystal. 

## 4. Summary

This paper reviewed the contamination phenomena of the impurities produced during CZ and directional solidification crystal growth methods using experimental and numerical approaches. CO contamination in the CZ method is a result of the reaction between SiO and C generated at the surface of the heaters and at the interface between the SiO_2_ crucible and the carbon holder. The source of O and N contamination in the UD furnace is the interface between the Si melt and the SiO_2_ crucible covered with Si_3_N_4_.

## Data Availability

Not applicable.
